# Successful treatment with bortezomib-containing regimen of primary plasma cell leukemia: a case report

**DOI:** 10.11604/pamj.2020.36.15.21717

**Published:** 2020-05-12

**Authors:** Mohammad Bader Obeidat, Ali Mohammad Al-Swailmeen, Abdulmajeed Mohammad Arabeat, Ayman Sulaiman Abukamar

**Affiliations:** 1 Department of Medicine, Hematology and Oncology Unit, King Hussein Medical City, Amman, Jordan

**Keywords:** Plasma cell leukemia, multiple myeloma, bortezomib

## Abstract

Plasma cell leukemia represents the most aggressive form of plasma cell dyscrasia. We report a 67-year old male with no previous medical illnesses presented with anemic symptoms. Blood film revealed 35% circulating plasma cells, bone marrow biopsy showed plasma cells constituting 85%. Diagnosis of primary plasma cell leukemia was completed. Induction chemotherapy with bortezomib, doxorubicin, and dexamethasone was started. After the first cycle, plasma cells in peripheral blood disappeared. The patient had complete remission at evaluation after the third cycle. Re-evaluation after the sixth cycle showed that he maintained remission. As he was non-transplant eligible, he was we kept on maintenance bortezomib. Twenty-four months after the diagnosis, the patient remains in remission.

## Introduction

Plasma cell leukemia (PCL) represents the most aggressive form of plasma cell dyscrasia. The incidence is thought to be less than 1 case/million [1]. It is divided into primary plasma cell leukemia (pPCL) which constitutes about 60% of cases, which occurs de novo without evidence of the previous history of plasma cell disorder. While secondary plasma cell leukemia (sPCL) represent progression and leukemic transformation in a patient previously diagnosed with multiple myeloma (MM). The prognosis of pPCL is poor with median survival reported being 2 to 8 months [2]. The diagnosis requires peripheral plasma cells of more than 2×10^9^/L or a greater than 20% of leukocyte count [3]. We present a patient diagnosed with pPCL who achieved complete remission after induction chemotherapy with bortezomib, doxorubicin and dexamethasone followed by maintenance bortezomib as he was non-transplant eligible.

## Patient and observation

**Figure 1:**
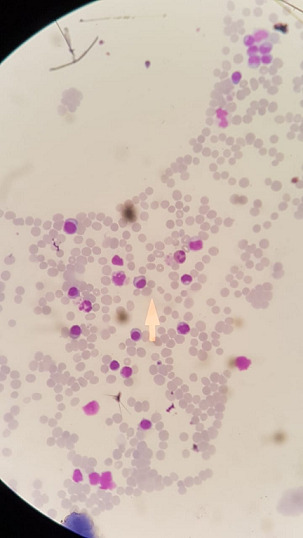
Blood film shows 35% circulating plasma cells in peripheral blood

A 67-year old male previously healthy was admitted in January 2018 to our hospital with fatigue, dyspnea on effort and multiple bone pains. The physical examination was unremarkable except for pallor and mild splenomegaly. Initial laboratory investigations showed macrocytic anemia with a hemoglobin of 9.9 g/dl, white blood cell count of 19.4x10^3^/μl and platelet count was 77x10^3^/μl. Erythrocyte sedimentation rate (ESR) was 60 mm/hr. Electrolyte panel revealed hypercalcemia (10.8 mg/dl) (reference 8.5-10.6) in the setting of slightly elevated creatinine level (1.7 mg/dl). Liver enzymes and bilirubin were normal. Positron emission tomography (PET) imaging showed moderate in homogeneous increased bone marrow activity throughout the whole skeleton associated with multiple insufficiency fractures in multiple thoracolumbar vertebrae. Blood film revealed leucoerythroblastic anemia and leukocytosis with 35% circulating plasma cells in atypical and immature forms (Figure 1). Bone marrow aspirate showed hypercellular bone marrow due to infiltration by atypical plasma cells constituting 60-70% of bone marrow cells. Core bone marrow biopsy revealed a markedly increased plasma cell, constituting 85% of the marrow elements as evidenced by CD138 immunostaining and showed kappa light chain restriction and all bone marrow elements were quantitatively decreased. Further blood investigations revealed the following: serum protein electrophoresis was normal with no evidence of monoclonal proteins, beta-2 microglobulin was within normal limits, albumin 3.37 gm/dl (reference 3.5-6.0), total protein 5.22 g/dl (reference 4.05-8.0). Echocardiogram showed normal left venticular systolic function and viral markers were negative. Based on the clinical and laboratory findings, the patient was diagnosed as having primary plasma cell leukemia. Supportive therapy was started immediately and consisted of hydration, allopurinol, pamidronate, antimicrobial prophylaxis, anti-viral medications, and prophylactic anticoagulation. Induction chemotherapy with VAD protocol: bortezomib 1.3 mg/m^2^ (days 1, 4, 8, and 11), doxorubicin 9 mg/m^2^ [4days 1 to 4], dexamethasone 20 mg (days 1, 4, 8, and 11) was initiated. The treatment was repeated every three weeks throughout six cycles. After the first cycle, the patient's general health and well-being dramatically improved with the gradual recovery of white blood cells, hemoglobin, platelets, and creatinine. The plasma cells in peripheral blood disappeared. He tolerated treatment well with minimal side effects. Evaluation after the third cycle in April 2018; blood film showed normochromic, normocytic red blood cells and no evidence of plasma cells. A repeated bone marrow aspirate and biopsy showed plasma cells constitute up to 5%-8% as evident by CD138. The patient had complete remission (CR) based on the International Myeloma Working Group (IMWG) criteria [4]. After the fifth cycle, the patient was admitted with neutropenic fever and was treated with antibiotics and filgrastim, he was stabilized after a few days and was given the sixth cycle with a delay of one week. Re-evaluation after the sixth cycle in August 2018: blood morphology showed no evidence of plasma cells. Bone marrow assessment showed no morphologic or cytogenetic evidence of plasma cells. As he was non-transplant eligible, we maintained him on bortezomib dose of 1.3 mg/m^2^ (days 1, 8, 15, and 22) and dexamethasone 20 mg (days 1, 8, 15, and 2) of a 35-day cycle. In January 2019 he complained of peripheral neuropathy (grade 2, WHO classification), which is a well-documented side effect of bortezomib therapy. So, the frequency of bortezomib maintenance was decreased to 1.3 mg\m^2^ every 2 weeks and dexamethasone was stopped. Twenty-four months after the PCL diagnosis and at the time of preparing the article the patient remains in CR on maintenance bortezomib every 2 weeks.

## Discussion

PCL was first described in 1906 by Professor Gluzinski and Dr. Reichenstein who established the first diagnostic criteria for PCL: plasma cells circulating in the peripheral blood (CPCs) of more than 2×10^9^/L or a greater than 20% of the peripheral blood leukocyte count. This definition has lots of drawbacks; first, it requires a high number of CPCs in peripheral blood detected with conventional count for diagnosis. Many patients with a level <20% threshold may have similar outcomes to those with = 20% CPCs [5]. Second, the diagnosis of PCL is operator dependant; it needs for hematologist or pathologist to count CPC by light microscope. Thus, diagnosis with immunophenotyping by Multiparameter Flow Cytometry is warranted. Lastly, the definition didn't differentiate between pPCL which occurs de novo and sPCL which consider being a leukemic progression of MM. Therefore, the International Myeloma Working Group (IMWG) announced for studies to revise criteria for the diagnosis of PCL [6]. There is no standard therapeutic strategy for the treatment of PCL as most of the information about PCL has been extrapolated from case reports, case series, and retrospective reports. There were very few prospective studies that evaluated the best management for PCL. The main goals of treatment are to eradicate malignant plasma cells, reduce the burden of disease, reverse end-organ damage, and prevent early mortality and to improve survival. The treatment plans for PCL follow the guidelines of the management of MM. Induction chemotherapy includes various bortezomib based regimens. In the transplant-eligible patients, this is followed by high-dose chemotherapy with autologous stem cell transplant aiming to prolong remission. This could be followed by maintenance with novel agents. While in patients who are considered to be non-transplant eligible whether because of advanced age or comorbidities the benefits of maintenance treatment after induction of remission is highlighted, precautions should be made in these patients to prevent infections with prophylaxis antibiotics, antiviral and antifungal because of their remarkably impaired immune system. Also, prophylaxis with allopurinol is essential because these patients are prone to tumor lysis syndrome [7]. Bortezomib-based regimens show promise as induction regimens for PCL. A large multicenter retrospective survey conducted by the GIMEMA group in 2012 focused on pPCL patients who had received bortezomib exclusively as frontline therapy. Twenty-nine patients with pPCL were collected. An overall response rate of 79% was observed, after a median follow-up of two years, the progression-free survival (PFS) was 55% [8]. Another multicenter retrospective study included 73 patients with pPCL, different regimens were examined, and the best outcome was for patients who treated with bortezomib-based regimens followed by autologous stem cell transplantation (ASCT) [9]. Based on the above results we started induction chemotherapy with a bortezomib-based regimen. ASCT has been widely used in the treatment of transplant-eligible patients with pPCL aiming to achieve a deep response and prolong remission. Patients with pPCL not eligible for ASCT, the induction therapy should be followed by maintenance regimens which can include either bortezomib or lenalidomide or both [10]. So, our patient was kept on maintenance bortezomib till progression.

## Conclusion

Our case demonstrates a potentially curative strategy (and relatively well-tolerated) for newly diagnosed transplant-ineligible pPCL with a combination of bortezomib, doxorubicin, and dexamethasone in the induction phase, followed by maintenance bortezomib.

## Competing interests

The authors declare no competing interests.
